# Normothermic Ex Situ Machine Perfusion of Vascularized Composite Allografts with Oxygen Microcarriers for 12 Hours Using Real-Time Mitochondrial Redox Quantification

**DOI:** 10.3390/jcm12206568

**Published:** 2023-10-17

**Authors:** Valentin Haug, Yifeng Peng, Bianief Tchiloemba, Alice T. Wang, Florian Buerger, Padraic Romfh, Ulrich Kneser, Brian D. Polizzotti, Bohdan Pomahac

**Affiliations:** 1 Division of Plastic Surgery, Brigham and Women’s Hospital, Harvard Medical School, Boston, MA 02115, USA; vfm.haug@gmail.com (V.H.);; 2 Department of Hand, Plastic and Reconstructive Surgery, Microsurgery, Burn Trauma Center, BG Trauma Center Ludwigshafen, University of Heidelberg, 67071 Ludwigshafen, Germany; 3 Department of Cardiology, Boston Children’s Hospital, Harvard Medical School, Boston, MA 02115, USA; 4 Division of Plastic Surgery, Department of Surgery, University of Calgary, Calgary, AB T2N 4N1, Canada; 5 Harvard Medical School, Boston, MA 02115, USA; 6 Department of Pediatrics, Boston Children’s Hospital, Harvard Medical School, Boston, MA 02115, USA; 7 Pendar Technologies, Cambridge, MA 02138, USA; 8 Division of Plastic and Reconstructive Surgery, Yale University School of Medicine, New Haven, CT 06510, USA

**Keywords:** vascularized composite allograft, ex situ perfusion, oxygen microcarriers, real-time mitochondrial redox state quantification, Raman spectroscopy, ex situ tissue preservation

## Abstract

Background: Normothermic ex situ perfusion of vascularized composite allografts (VCAs) necessitates high oxygen demand and, thus, increased metabolic activity, which, in turn, requires the use of blood-based perfusion solutions. However, blood-derived perfusates, in turn, constitute an antigenic load. To circumvent this immunogenic problem, we used a perfusate enriched with acellular dextrane oxygen microcarriers to perfuse rat hindlimbs. Methods: Rat hindlimbs (n = 11) were perfused with either (non-), oxygenated dextrane-enriched Phoxilium, or Phoxilium enriched with dextrane oxygen microcarriers (MO_2_) for 12 h at 37 °C or stored on ice. Oxygenation of the skeletal muscle was assessed with Raman spectroscopy, tissue pO_2_-probes, and analysis of the perfusate. Transmission electronic microscopy was utilized to assess the ultrastructure of mitochondria of the skeletal muscle. Results: For all evaluated conditions, ischemia time until perfusion was comparable (22.91 ± 1.64 min; *p* = 0.1559). After 12 h, limb weight increased significantly by at least 81%, up to 124% in the perfusion groups, and by 27% in the static cold storage (SCS) group. Raman spectroscopy signals of skeletal muscle did not differ substantially among the groups during either perfusion or static cold storage across the duration of the experiment. While the total number of skeletal muscle mitochondria decreased significantly compared to baseline, mitochondrial diameter increased in the perfusion groups and the static cold storage group. Conclusion: The use of oxygen microcarriers in ex situ perfusion of VCA with acellular perfusates under normothermic conditions for 12 h facilitates the maintenance of mitochondrial structure, as well as a subsequent recovery of mitochondrial redox status over time, while markers of muscle injury were lower compared to conventional oxygenated acellular perfusates.

## 1. Introduction

Minimizing ischemic injury of vascularized composite allografts (VCAs) after surgical separation from their physiological blood supply is crucial for the reduction of ischemia–reperfusion injury (IRI) after vascularized composite allotransplantation or replantation. Under optimal conditions of conventional preservation, the allowable ischemia time for VCAs is not more than six hours, as skeletal muscle is vulnerable to IRI [1].

Ex situ machine perfusion (EMP) facilitates the supply of oxygenated perfusates with essential nutrients and electrolytes for VCAs, exceeding the viable preservation time of static cold storage (SCS) as a preservation method by a multitude, which has been the gold standard preservation method for solid organ transplantation and preservation of amputated limbs [2,3]. These findings have been confirmed in small and large animal experiments [3,4,5]. Different EMP strategies evolved, including hypo-subnormothermic and normothermic perfusion. While hypothermic and subnormothermic perfusions decrease the tissues’ metabolic demand, normothermic perfusion allows for viability testing of skeletal muscle and nerves during ex situ perfusion prior to transplantation. As a tissue’s metabolic activity is temperature-dependent and decreases by half for every 10 °C reduction, near-normothermic EMP mimics in vivo conditions, which creates the necessity for blood-based perfusates [6]. However, those are immunogenic, potentially infectious, require specialized storage in a blood bank, and have a short shelf-life [7,8]. Acellular perfusates do not offer a sufficient oxygen-carrying capacity for a satisfactory supply of VCAs with oxygen in normothermic settings. One potential approach to resolve the time-sensitive issue of hypoxia in a normothermic amputated limb is the intravascular administration of artificial oxygen carriers, which can deliver a five-fold amount of oxygen compared to a human red blood cell, adjusted by weight [9].

In this study, we report our strategy to enhance the beneficial effects of normothermic EMP with rapid-release microparticles for VCAs in a 12 h rat hindlimb perfusion model to reduce the devastating effects of deficient oxygen delivery in skeletal muscle.

## 2. Methods

### 2.1. Animal Protocol

**Figure 1:**
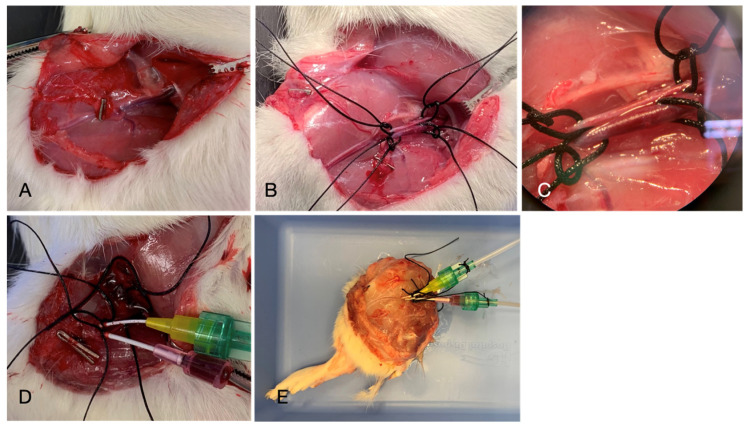
(**A**) Clipping of the superficial epigastric artery after inguinal vascular access. (**B**) Dissection of the femoral artery and vein. (**C**) Microsurgical preparation for the arteriotomy and venotomy with ligatures. (**D**) Cannulation of femoral artery and femoral vein with #3 French venous catheters via Seldinger method. (**E**) Completely cannulated limb flushed with heparinized saline.

This experimental study was approved by the Institutional Review Board and the IACUC (Protocol Number: BCH-18-08-3713R). In this non-survival experiment, male Sprague-Dawley rats of at least 6 weeks of age were used. After weighing the experimental animal, they were put in an induction chamber with isoflurane 2% for 4 min, followed by an intraperitoneal injection of ketamine and xylazine. Sedated rodents were placed on a nose cone with isoflurane to maintain the anesthesia. Electric heating pads with a rectal-probe feedback loop were utilized to prevent hypothermia in the experimental animal and maintain body temperature at 37 °C. After preoxygenation, animals were intubated and ventilated on 30% oxygen during baseline measurements for 15 min. Femoral vasculature was exposed through an inguinal incision (Figure 1). After microsurgical dissection of the femoral artery and vein below the inguinal ligament, vessels were prepared for ligature, and 100 IU of heparin was administered intraperitoneally 10 min before ligation of the vessels. Baseline Raman spectroscopy measurements were performed on the adductor muscle after dissecting the overlaying fascia. Artery and vein were incised and cannulated with #3 French venous catheters. The artery was immediately flushed with heparinized saline to prevent intravascular clotting. 

Animals were euthanized by anesthetic overdose (4–5% isoflurane) and cardiac puncture. Once the animal’s death had been confirmed, transfemoral amputation was performed, and the amputated limb was weighed. The time of ischemia and confirmed death until the beginning of the perfusion or cooling, respectively, were recorded. 

### 2.2. Experimental Setup

Through the catheter, the limb was connected to the perfusion setup via luer lock connectors. Perfusion with a continuous flow rate of 1 mL/min, as assessed in previous experiments as the physiological flow of the femoral artery, was started with (I) oxygenated modified, dextrane-enriched Phoxilium, (ii) non-oxygenated dextrane-enriched Phoxilium, and (iii) Phoxilium enriched with oxygen microcarriers. A detailed overview of the experimental groups can be seen in Table 1.

The detailed composition of the perfusion solution was published in our work in 2020 [5]. Briefly, the balanced electrolyte solution Phoxilium™ (Baxter, Deerfield, IL, USA) was modified to tailor it to the specific requirements of VCA machine perfusion. Dextran with 40,000 Dalton served as a colloid. Fifty percent Dextrose (Hospira, Inc., Lake Forest, IL, USA) was added to a concentration of 0.1%. Insulin R (Lilly USA, LLC, Indianapolis, IN, USA) was added to a concentration of 0.0075%. Further additives were 125 mg of methylprednisolone and 2500 units/L of heparin (both Fresenius Kabi, Lake Zurich, IL, USA).

Calibrated Baxter i.v. pumps (Baxter, Deerfield, IL, USA) were used to transport the preheated perfusate into the limb. Pressure was assessed through an in-line luer-lock pressure sensor (Pendar, Cambridge, MA, USA).

Limbs of the control group were stored in an ice slurry at 4 °C (static cold storage (SCS)) for the entire duration (12 h) of the experiment.

Temperature, pressure, and tissue pO_2_ were continuously recorded using LabChart Pro-8 software (ADInstruments) software. Perfusate samples were drawn from the cannulated femoral vein.

Osmotic pressure of the perfusate was measured with a vapor pressure osmometer (Wescor Vapro, Logan Utah, UT, USA) and averaged around 305 mOsmol/kg.

### 2.3. Oxygen Microcarriers

The fabrication of polymeric microbubbles (mean size = 5 μm) was thoroughly reported in our previous work [10].

Briefly, nanoprecipitation of biocompatible dextran, as the base of the polymer, was utilized to produce stable microbubbles. The 100% oxygen loading of oxygen microcarriers was achieved by passively purging the headspace of microbubble suspension in 10% dextrose solution, as measured with a dissolved pO_2_ > 750 mmHg. The oxygenated microbubbles can be stored in a capped syringe to maintain oxygen content and are stable for months at room temperature.

### 2.4. Raman Spectroscopy

A Resonance Raman Spectrometer was used to quantify the mitochondrial redox state in the skeletal muscle of the limb in real time, as described previously for myocardial muscle [11].

Briefly, a single-mode laser light source with a specific wavelength of 441 nm excites specific molecular bonds to obtain a spectroscopical read-out. In this study, we used resonance Raman reduced mitochondrial ratio (3RMR) to quantify the mitochondrial redox state of the skeletal muscle within the limb during the ex situ perfusion.

Before amputation, baseline measurements of the physiologically perfused skeletal muscle were performed to generate a spectral library of the different components.

### 2.5. Ultrastructural Analysis

Skeletal muscle samples were harvested before amputation of the hindlimb directly after amputation, as well as at 6 and 12 h post-amputation. Samples were fixed in 2.5% glutaraldehyde, 1.25% PFA, and 0.03% picric acid in 0.1 M sodium cacodylate buffer (pH 7.4) overnight at 4 °C. Samples were then washed with 0.1 M phosphate buffer, post-fixed with 1% OsO4 dissolved in 0.1 M phosphate-buffered saline (PBS) for 2 h, dehydrated in ascending gradual series (50–100%) of ethanol, and infiltrated with propylene oxide. Samples were embedded using the Poly/Bed 812 kit (Polysciences) according to the manufacturer’s instructions. After pure fresh resin embedding and polymerization in a 65 °C oven (TD-700, DOSAKA, Kyoto, Japan) for 24 h, sections of approximately 200–250 nm thickness were cut and stained with toluidine blue for light microscopy. Sections of 70 nm thickness were double-stained with 6% uranyl acetate (EMS, 22400; Hatfield, PA, USA) for 20 min and lead citrate (Fisher) for 10 min for contrast staining. The sections were cut using Reichert Ultracut-S/LEICA EM UC-7 (Leica, Deer Park, IL, USA) with a diamond knife (Diatome Ltd., Nidau, Switzerland) and transferred onto copper and nickel grids. All the sections were observed by transmission electron microscopy (JEOL 1200EX, Bronx, NY, USA) at an acceleration voltage of 80 kV. All steps, including image acquisition, were performed in a blinded manner by independent persons (F.B., A.W., B.T.). Image analysis was performed using the open-source application Image J 1.52 k (Wayne Rasband, NIH, USA).

### 2.6. Perfusion Solution Sample Analysis

Collection from the venous cannulation was performed at timepoint 0, directly after starting the perfusion, and then every two hours until timepoint 12 h. Perfusion solution samples were analyzed for markers of muscle injury, such as CK, LDH, and myoglobin. Additionally, blood gas analysis was performed for pH, pO_2_ and pCO_2_, potassium (K+), glucose, and lactate no later than 1 h after collection. Blood gas analysis was performed with an 837 Flex Radiometer (Radiometer Inc., Brea, CA, USA). Analysis of myoglobin was carried out with Cobas e602, lactate analysis with Cobas c702, and LDH and CK with Cobas c502 (Roche Diagnostics, IL, USA), as previously published [2].

### 2.7. Statistical Analysis

For the comparison between multiple groups, a single ANOVA test was performed.

Differences amongst multiple groups and different timepoints were calculated in a mixed effect model or a two-way ANOVA. To account for multiple comparisons, *p*-values were adjusted via Tukey post hoc correction. A *p*-value of <0.05 was considered statistically significant. Results are shown as the mean with standard deviation. All statistical analyses and visualizations were carried out using GraphPad Prism version 9.4.1 for MacOS (GraphPad Software, La Jolla, CA, USA).

## 3. Results

### 3.1. Experimental Animal Data

Experimental animals (n = 11) were randomly assigned to the four experimental groups (Table 1). The average weight was 511 ± 47 g, without significant differences between groups (*p* = 0.6426). 

**Figure 2:**
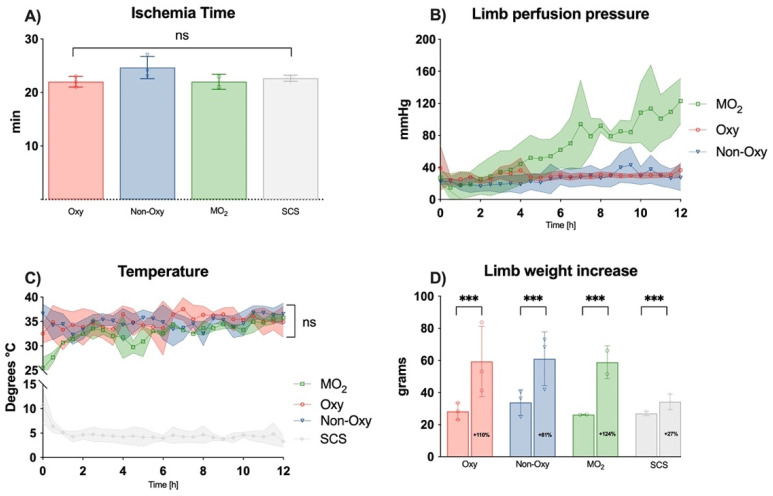
(**A**) Ischemia time of the different groups in minutes; ns = not significant. (**B**) Limb perfusion pressure in mmHg over time. After correction for multiple comparisons (multiple groups, multiple timepoints), no significant differences were detected among all groups. (**C**) Temperature in degrees Celsius over time. No significant differences were detected among the perfusion groups, as perfusion was executed under normothermic conditions. (**D**) Limb weight showed significant changes from timepoint 0 h (left column) to timepoint 12 h (right column) among all experimental groups [*** *p* = 0.002]; notably, the weight gain was higher in the perfusion groups.

Ischemia time, precisely the time from ligature of the femoral vessels to completion of the cannulation and the first flush, was comparable, with a mean of 22.91 ± 1.64 min (*p* = 0.1559) among the different groups (Figure 2A).

### 3.2. Machine Data

Perfusion pressure started to increase in the MO_2_ group after 6 h and showed to be substantially higher compared to the other perfusion groups (Figure 2B). Temperatures in the perfusion groups were consistent and not significantly different within the perfusion groups (Figure 2C). Obviously, the temperature in the SCS group was significantly lower, averaging 4.67 ± 1.81 °C, with a rapid drop within the first hour of storage. The mean temperature in the perfusion groups was 34.32 ± 2.56 °C. 

Over the course of 12 h perfusion, limb weight increased significantly, by at least 81% (Oxy group) and up to 124% (MO_2_ group) in the perfusion groups and by 27% in the SCS group, as depicted in Figure 2D. Differences were not significant among the perfusion groups. 

### 3.3. Ultrastructural Analysis

**Figure 3:**
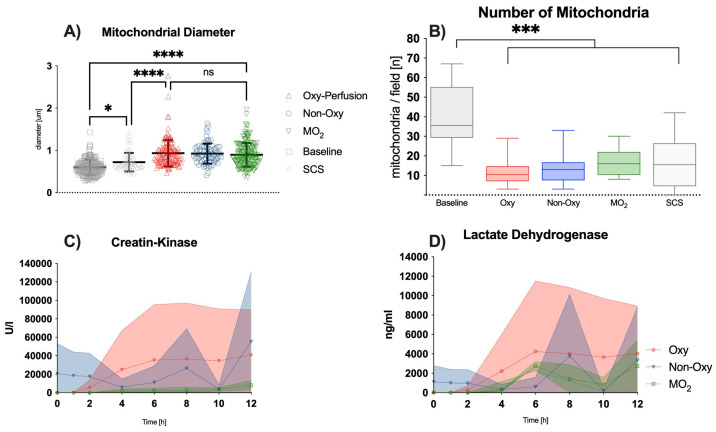
(**A**) Mitochondrial diameter (um) at timepoint 12 h; significant increase compared to baseline in the SCS group [* *p* = 0.0009] and compared to all perfusion groups [**** *p* < 0.0001]; significantly lower diameter in the SCS group compared to all perfusion groups [**** *p* < 0.0001]; ns = not significant. (**B**) Number of mitochondria after 12 h of perfusion [*** *p* < 0.0001]; for better readability, brackets between the perfusion groups indicating “ns” were omitted. (**C**) Levels of creatine kinase over 12 h; no significant differences among the perfusion groups were noted. (**D**) Levels of lactate dehydrogenase over 12 h; no significant differences among the perfusion groups were noted.

The average mitochondrial diameter in the skeletal muscle after normothermic perfusion of 12 h was significantly increased in comparison to skeletal muscle tissue at baseline. However, after 12 h of static cold storage, the average diameter of mitochondria was significantly smaller compared to the perfusion groups (*p* < 0.0001) and skeletal muscle tissue at timepoint zero (*p* = 0.0009) (Figure 3A). Among perfusion groups, significant differences could be observed.

The total number of mitochondria in skeletal muscle tissue samples significantly decreased over the course of 12 h in the perfusion groups and the SCS group (all *p* < 0.0001) when compared to the baseline at timepoint 0 before amputation. Interestingly, no substantial differences were detected, neither among the perfusion groups nor in the SCS group (Figure 3B).

**Figure 4:**
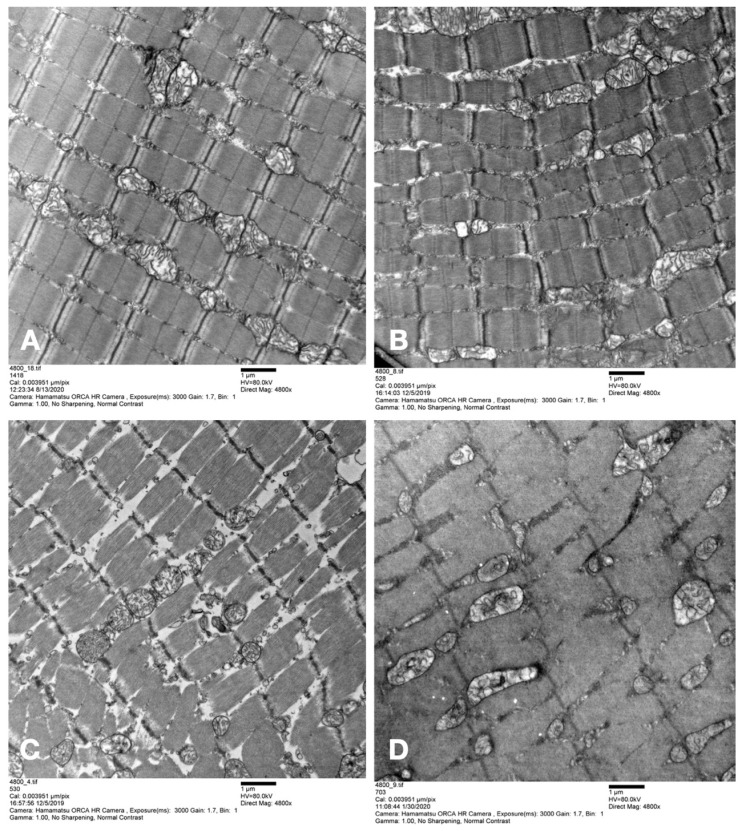
Representative results of the transmission electron microscopy ultrastructural analysis of the skeletal muscle biopsies after 12 h of perfusion or static cold storage, respectively. (**A**) MO_2_ group. (**B**) Oxygenated perfusate. (**C**) Non-oxygenated perfusate. (**D**) Static cold storage.

Representative TEM-sections of skeletal muscle biopsies after 12 h of ex-situ machine perfusion or SCS are depicted in Figure 4A–D.

### 3.4. Clinical Chemistry Analysis of the Perfusate

**Figure 5:**
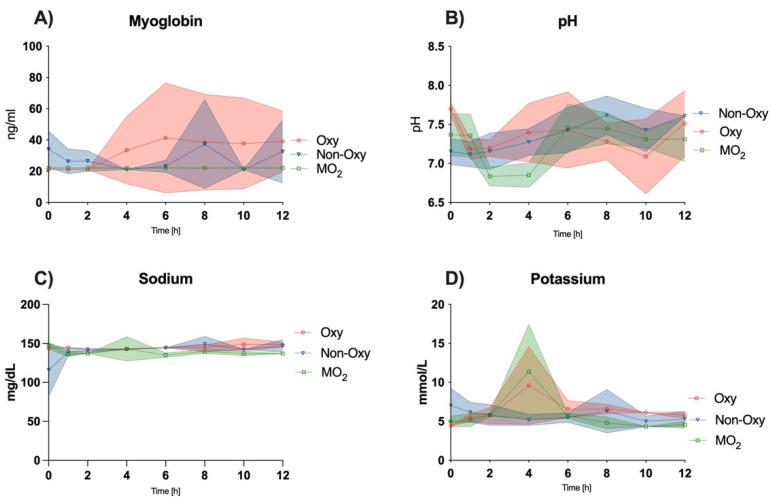
Analysis of the perfusion solutions for (**A**) levels of myoglobin (ng/mL) over 12 h; (**B**) pH levels over 12 h; (**C**) sodium levels (mg/dL) over 12 h; (**D**) potassium levels (mmoL/L) over 12 h. Statistical differences were not observed in (**A**–**D**).

Perfusate analysis showed an overall increase in the values of creatine kinase (Figure 3C) and lactate dehydrogenase (Figure 3D) over time, while myoglobin levels were stable for 12 h (Figure 5A).

### 3.5. Blood Gas Analysis of the Perfusate

**Figure 6:**
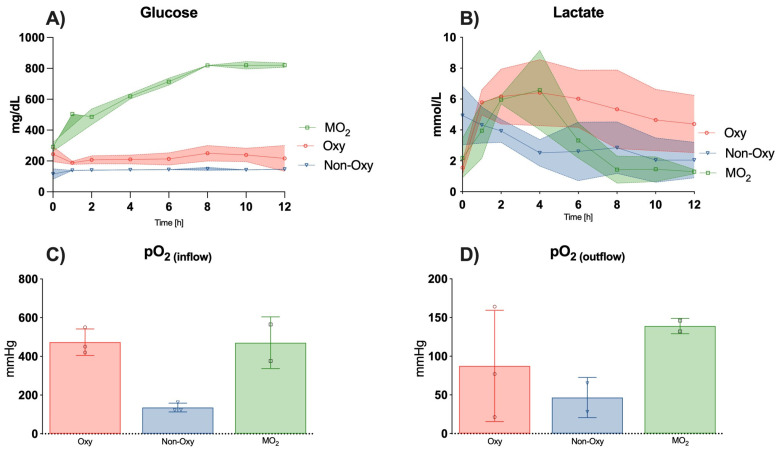
(**A**) Continuous levels of glucose in the perfusate in mg/dL, with significantly higher glucose levels in the MO_2_ group as compared to the group with the oxygenated and non-oxygenated perfusate. (**B**) Lactate levels in mmol/L. Significant differences were not observed. (**C**) Levels of pO_2_ in mmHg before entering the limb. Significant differences were observed when comparing the non-oxygenated perfusate with the oxygenated perfusate (*p* < 0.0001) and the MO_2_ group (*p* = 0.0002). (**D**) Levels of pO_2_ in mmHg exiting the limb.

The pH levels were consistent throughout the experimental course, appearing increasingly physiological after 6 h, after an initial decrease to acidic values for the first 4 to 6 h in all groups (Figure 5B). Sodium levels averaged around physiological values of 141.5 ± 9.8 mg/dL amongst all perfusion groups (Figure 5C), while potassium level measurements were in hyperkalemic ranges, with mean values of 6.28 ± 2.1 in the Oxy group, 5.76 ± 1.32 in the Non-Oxy group, and 5.84 in the MO_2_ group. Notably, potassium levels peaked in the Oxy group and the MO_2_ group at 4 h, then decreased to levels observed at timepoint 0 (Figure 5D). Glucose levels were lowest in the Non-Oxy group, followed by the Oxy group. In the MO_2_ group, a continuous increase in glucose levels was measured over the course of 12 h (Figure 6A), with significantly higher values as compared to the Oxy (*p* = 0.0010) and Non-Oxy (*p* = 0.0041) groups.

Lactate levels surged in the Oxy and MO_2_ groups for 4 h after the start of the perfusion, then decreased minimally in the Oxy group and dropped to lower values in the MO_2_ group as compared to the Non-Oxy group after 8 h (Figure 6B). Physiological values were only detected and maintained in the MO_2_ group, with mean values below 2 mmol/L after 8 h of perfusion. 

Before perfusing the limb, perfusate samples of the MO_2_ group and the Oxy group showed comparable values of 470.55 ± 133.57 mmHg or 473.33 + 68.10 mmHg, respectively. As expected, values were significantly lower in the Non-Oxy group, with a mean of 135.77 ± 22.74 mmHg (*p* = 0.0002) (Figure 6C). Oxygen outflow was measured over the course of the experiment and showed a gradual increase in the Non-Oxy group, almost doubling the mean value after starting the perfusion from 73 mmHg to 140 mmHg; interestingly, values in the Oxy group were consistent, with a slight increase from a mean of 106 mmHg to 140 mmHg. The biggest increment was measured in the MO_2_ group, with means of 123 mmHg at the beginning of the perfusion and 205 mmHg after 12 h of perfusion. Mean levels of pO_2_ outflow did not show significant differences (Figure 6D).

## 4. Tissue Oxygenation

Raman Spectroscopy and Tissue pO_2_

**Figure 7:**
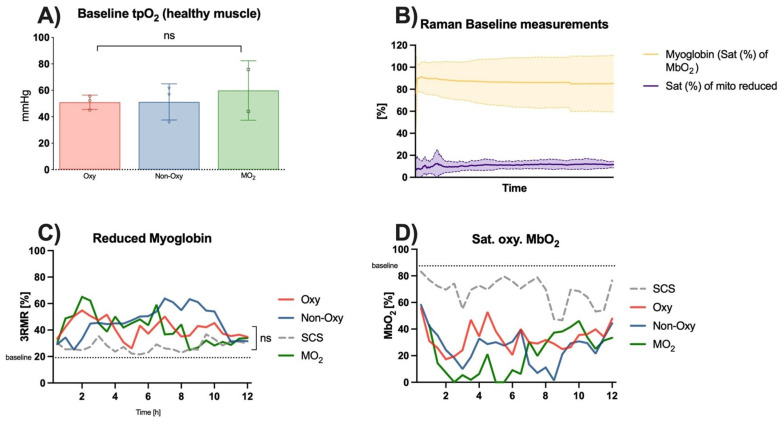
(**A**) Levels of tissue pO_2_ in healthy muscle in mmHg; ns = not significant. (**B**) Exemplary Raman spectroscopy measurements in healthy muscle before amputation. (**C**) Levels of 3RMR (%); no significant differences were noted. (**D**) Levels of MbO2 (%); no significant differences were noted.

Measurements of healthy skeletal muscle perfused in the anesthetized rat showed a mean baseline tissue pO_2_ value of 53.3 ± 12.3 mmHg prior to cannulation and perfusion (Figure 7A). Raman spectroscopy showed a saturation of the oxygenated myoglobin of 87.47 ± 11.6% and a saturation of the reduced myoglobin of 19.12 ± 5.51% (Figure 7B).

3RMR signals of skeletal muscle during either perfusion or static cold storage over 12 h did not show significant differences within the groups. Values in the SCS group were the closest to the aforementioned baseline values. Values of reduced myoglobin in the perfusion groups peaked after 2 h of the experiment in the MO_2_ group and the Oxy group and then gradually decreased to twofold baseline values after 12 h. Values in the Non-Oxy group peaked after 7–9 h of perfusion and then decreased as well towards the end of the 12 h (Figure 7C). Statistical analysis did not show substantial differences among the different groups.

Consistent with these results, saturation of oxygenated myoglobin showed a sudden decrease in the values in all groups after the first 2–3 h of the experiment. Apart from peaks around hour 4 of perfusion, the saturation of oxygenated myoglobin remained low, particularly in the Oxy group and the MO_2_ group. These groups showed a recovery from hour 6 until the end of the experiment. Values in the Non-Oxy group only decreased between hours 8–10. The trend in the values in the static cold storage group was similar to that in the Non-Oxy group; however, they were closer to baseline values (Figure 7D).

**Figure 8:**
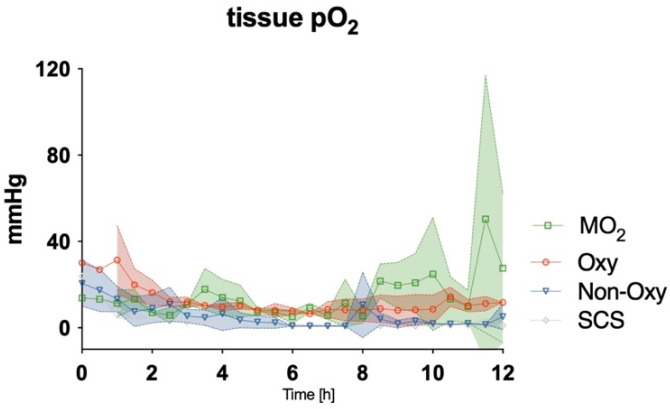
Levels of tissue pO_2_ in skeletal muscle in mmHg over the course of 12 h. Significant differences between all groups were not detected.

After detaching the cannulated limb and either storing it on ice or attaching it to the perfusion devices, levels of tissue pO_2_ were assessed with an oxygen probe. A gradual decrease was observed in all groups during the first 2–3 h of the experiment. While values for tissue pO_2_ remained low in the SCS group and the Non-Oxy group for the entire duration of the procedure, tissue pO_2_ in the Oxy group and the MO_2_ group remained at circa 20% of the baseline values from hour 3 to 8. After hour 8, a gradual increase in tissue pO_2_ could be observed in the MO_2_ group, up to 50% of baseline values (Figure 8). Statistical differences were not observed among the groups. 

## 5. Discussion

The use of dextrane polymer oxygen microcarriers for the normothermic ex situ perfusion of rat hindlimbs proved to be beneficial, with (i) higher levels of tissue pO_2_ and (ii) lower levels of lactate after 8–12 h as compared to groups in which acellular perfusion solutions were used as perfusate, while ischemia times and temperatures were comparable. Mitochondrial diameter remained comparable to the values in the other perfusion groups after 12 h of perfusion and was significantly higher as compared to the static cold storage group, while the total number of mitochondria decreased significantly in all ex situ perfusion groups. 

Raman spectroscopy of the skeletal muscle mitochondria redox state during the ex situ perfusion or static cold storage did not show significant differences between the different preservation techniques. After 8 h of perfusion, however, a trend towards values closer to baseline could be observed in the MO_2_ group, both for reduced and oxygenated mitochondria. Given the amount of scattered data and the multitude of measurements, statistical differences between the perfusion groups were not found.

In previous studies, we have demonstrated the feasibility of ex situ perfusion of VCAs with acellular solutions under hypothermic conditions [2,5]. Given the tissue’s increased metabolic demand under normothermic conditions, we are now able to demonstrate non-inferiority towards conventional tissue conservation methods, such as static cold storage and hypothermic ex situ perfusion. Normothermic perfusion enables the testing of skeletal muscle during the experiment and directly prior to transplantation. 

Over the course of the experiment, a substantial weight gain was observed in all perfusion groups, which is in accordance with previous animal experiments by our group in a large animal model [5]. Weight increase of the perfused limbs ranged from 84–121% and was similar to the results of other working groups with similar perfusion settings (110–130%) [12]. As shown in previous experiments, weight gain was linked to interstitial edema and was higher in groups with acellular solutions enriched with dextrane as compared to solutions with different colloids [5]. In a study with human allografts and a pressure-controlled perfusion model, only minimal weight gain was observed after 24 h with a colloidal perfusate (+4.3%) [2]. In the SCS group, the repeated irrigation of the extremity to prevent tissue from drying out might have led to an untypical weight gain. 

Flow rates of perfusion were comparable to physiologic conditions in resting muscle, as previously published, and in accordance with our preliminary measurements with microvascular flow probes [13,14,15]. Different research groups performed ex situ perfusion experiments with higher flow rates, such as 2.5 mL/min, and reported higher perfusion pressures of MO_2_ 100 mmHg in the femoral artery. Those values were not reached earlier than 6 h after perfusion in the MO_2_ group in our experimental setting, which might play a role in the formation of edema. 

In contrast to other publications reporting a subsequent increase in lactate levels within the perfusion solution in a hypothermic setting, our results demonstrate a decrease in lactate levels after a peak between hours 2–4 of perfusion in the MO_2_ group to physiological levels [16]. These findings are consistent with the trend shown by Gok et al. under near-normothermic conditions after perfusion for 6 h [15]. Araki et al. found lactate levels decreased after one hour of perfusion until hour 6 in rat hindlimbs under normothermic conditions with extracellular-trehalose-Kyoto (ETK) solution with and without hemoglobin vesicles [12]. Perfusate flow in the femoral artery was comparable at <1mL/min.

Potassium levels peaked after two hours of perfusion in the Oxy and MO_2_ groups and equilibrated at a level of mild hyperkalemia. Gok et al. reported similar values in a 6-h perfusion setting with hemofilter and an increase without hemofilter. 

Other markers of skeletal muscle cell injury, e.g., CK, LDH, and myoglobin, increased to a greater extent in the Oxy and Non-Oxy groups when compared to the MO_2_ group. Similar trends could be observed in a study by Herold et al., where LDH levels were found to rise and plateau after several hours of subnormothermic perfusion of adipofascial flaps in a rat model [17]. These trends are analogous to previous large animal and human studies under hypothermic conditions and near-normothermic conditions, respectively [2,5,18].

CO_2_ and O_2_ levels in the efflux appeared to be comparable to trends in the study by Araki et al. [12].

## 6. Limitations

Our study sheds new light on normothermic perfusion of VCAs in a small animal model using artificial oxygen microcarriers. The study’s limitations must be taken into consideration for the interpretation of our results. To our knowledge, this is the first study to evaluate the effects of normothermic perfusion with artificial oxygen carriers in an acellular perfusate on skeletal muscle and its mitochondria. However, the study’s sample size remains small due to the extensive and time-consuming experiments and the related high costs. As this study has a pilot character, no replantation and/or syngenic transplantation of the perfused limbs was performed to evaluate the effects of ischemia–reperfusion injury on the VCA. Prior to subsequent studies, the authors aim to analyze the effects of enriching acellular perfusates with oxygen microcarriers ex situ. Ensuing studies might aim to analyze the potential beneficial effects of perfusion with oxygen microcarriers after replantation to facilitate a valid assessment of the influence on IRI. 

## 7. Conclusions

We here demonstrate that the use of oxygen microcarriers in ex situ perfusion of VCAs with acellular perfusates under normothermic conditions for 12 h facilitates maintenance of mitochondrial structure and number, as well as subsequent recovery of mitochondrial redox status over time, while markers of muscle injury were lower compared to conventional oxygenated acellular perfusates.

## Figures and Tables

**Table 1:** Experimental groups: Oxy—oxygenated modified, dextrane-enriched Phoxilium; normothermic perfusion for 12 h. Non-Oxy—non-oxygenated dextrane-enriched Phoxilium; normothermic perfusion for 12 h. MO_2_—Phoxilium enriched with oxygen microcarriers; normothermic perfusion for 12 h. SCS—static cold storage for 12 h.

Groups	Oxy	Non-Oxy	MO_2_	SCS
Number (n)	3	3	2	3
Time (h)	12	12	12	12

## Data Availability

Data is contained within the article.

## Abbreviations

°C	degrees Celsius
CK	creatine kinase
CO_2_	carbon dioxide
EMP	ex situ machine perfusion
h	hour
HE	hematoxylin and eosin
IRI	ischemia–reperfusion injury
LDH	lactate dehydrogenase
min	minute
ml	milliliter
mmHg	millimeters mercury
MO_2_	oxygen microcarrier
MP	machine perfusion
ns	not significant
O_2_	oxygen
pH	potential of hydrogen
pO_2_	partial oxygen pressure
SCS	static cold storage
VCA	vascularized composite allograft
